# The Effect of an Iodinated Contrast Material Shortage on Ordering Patterns in the Emergency Department

**DOI:** 10.7759/cureus.32846

**Published:** 2022-12-22

**Authors:** Barry Hahn, Jerel Chacko, Mukund Mohan, Kurien Mathews, Josh Greenstein

**Affiliations:** 1 Emergency Medicine, Staten Island University Hospital, Staten Island, USA

**Keywords:** general radiology, emeregency medicine, medication shortage, contrast material, computed tomography

## Abstract

Introduction

Iodinated contrast media (ICM) is essential to emergency department care in differentiating and delineating life-threatening pathologies. In May 2022, due to the COVID-19 outbreak, there was an unprecedented disruption in the manufacturing of iodinated contrast. The primary goal of this study was to describe the effects of an ICM shortage on the ordering patterns of emergency medicine physicians.

Methods

This was a retrospective, observational study with a pre-/post-test design. The study included two 28-day periods. All subjects who underwent a CT were included in the study. The subgroup of patients who underwent a repeat CT with ICM contrast within 1-24 hours was identified.

Results

During the pre- and post-implementation study periods, 4,574 and 3,973 CT studies were performed. The median length of stay (p=0.013) and time to first CT (p<0.001) both decreased during the post-implementation period. During the post-implementation period, more non-contrast CTs were ordered (p<0.001). During the post-implementation period, there was an increase in non-contrast studies followed by a repeat study with contrast (p=0.003).

Conclusions

A global ICM shortage resulted in a shift in the ordering patterns of Emergency Medicine (EM) physicians. More non-contrast CT scans were ordered. However, there was also an increase in repeat imaging with ICM material.

## Introduction

Iodinated contrast media (ICM) is crucial for medical centers with emergency departments (ED), trauma centers, comprehensive stroke centers, and cardiac centers [1]. ICM, which includes oral and intravenous (i.v.) contrast material, is used to enhance the delineation of adjacent tissues and assess the integrity and function of tissues such as arteries, veins, and the blood-brain barrier [2]. ICM is essential to ED care in differentiating and delineating life-threatening pathologies. ICM shortages can impact timely and emergent diagnoses, morbidity, mortality, and missed findings [3].

In May 2022, during the initial phase of the COVID-19 outbreak, there was an unprecedented disruption in the manufacturing of ICM. GE healthcare’s primary production facility of ICM in Shanghai, China, had to halt production due to a government-mandated shutdown. Hospitals reliant on GE contrast media were the most impacted by the shortage. Many health systems and institutions created comprehensive workflows to mitigate the effect of these ICM shortages. These workflows focused on decreasing iodinated contrast media utilization across different specialties such as emergency medicine, radiology, neurology, cardiology, and vascular surgery [4]. These efforts have reported contrast utilization reduction rates of up to 85% [1, 5]. The American College of Radiology (ACR) has also published recommendations for the judicious use of ICM and alternative imaging studies. ACR recommendations include using alternative studies such as non-contrast computed tomography (CT), magnetic resonance imaging (MRI), ultrasound, alternate versions of contrast agents, prepackaging single-use vials, and other contrast products such as barium, and increased collaboration amongst medical specialties to reduce CT utilization [6].

At the outset of the ICM shortage, Northwell Health’s emergency medicine service line and the system’s chief medical office implemented mitigation and reduction strategies for ICM utilization. Furthermore, the health system supplied legal guidance for intricate case consultation and provided language surrounding non-contrast studies. Overall, there is a lack of literature on CT utilization patterns and potential adverse effects on ED operations in such a shortage. The primary goal of this study was to describe the effects of an ICM shortage on the ordering patterns of emergency medicine (EM) physicians.

## Materials and methods

Study design and setting

This was a retrospective, observational study with a pre-/post-test design. The study included two 28-day periods before and after ED staff received recommendations and guidelines on navigating the contrast shortage. The pre-implementation period used for comparison was April 11, 2022 - May 10, 2022, and the post-implementation period was May 11, 2022 - June 6, 2022. All patients who underwent CT scans were included in the study. Patients and providers were identified through an electronic health record (EHR), Allscripts Sunrise (Allscripts, Chicago, USA). The study was conducted at Staten Island University Hospital (SIUH), a member of Northwell Health, the largest academic health system in New York State. SIUH is a 700-bed specialized teaching hospital that occupies two geographically distinct clinical sites; SIUH -N is a 500-bed academic tertiary care center with 97,000 ED patient visits annually. SIUH -S is a 200-bed urban community hospital with 32,000 ED patient visits annually. These hospitals are located eight miles apart in Staten Island, New York, but share a single administration with unified oversight of operations, clinical quality, and doctor staffing. The local institutional review board reviewed the study.

Selection of participants

All subjects who underwent a CT were included in the study. The subgroup of patients who underwent a repeat CT with ICM contrast within 1-24 hours was identified. A repeat study was defined as any additional imaging occurring between 1-24 hours after the original study. This definition was implemented to avoid the inclusion of protocolized combination studies such as trauma or stroke protocols. Repeat studies were defined as additional imaging studies of the same body part with ICM after an initial non-contrast study. It was assumed that a repeat CT with ICM contrast within 1-24 hours could be attributed to a non-diagnostic non-contrast study. Patient demographics collected included age, gender, race, insurance status, arrival time to first CT performed, disposition type, and length of stay (LOS).

Intervention

On the evening of May 10, 2022, staff were notified of a global intravenous and oral contrast shortage. At that time, ED staff were encouraged to be mindful of the need for contrast use in all studies. Providers were urged to utilize clinical scoring systems, such as the Wells Score and Pulmonary Embolism Rule-out Criteria (PERC), whenever possible. Providers were also encouraged to consider ordering other studies that may offer similar diagnostic information (e.g., non-contrast CT, ultrasound, magnetic resonance imaging, or nuclear imaging). The system's Chief Medical Office created a guideline document to help guide clinical decision-making (Table 4, Appendices). If questions persisted regarding the appropriate study for a patient, staff were asked to call and speak with a subspecialty radiologist for a real-time radiological consultation.

In the ED, there was a temporary halt to ordering coronary computed tomography angiography (CCTA) studies. When appropriate, cardiology was consulted during regular working hours to arrange for possible outpatient management. Finally, an updated stroke imaging algorithm was created. In the first step of this new algorithm, non-contrast CTs continued to be ordered by ED clinicians. ED providers were then required to contact the on-call neurologist to confirm if computed tomography angiography (CTA) and perfusion studies were needed for the patient. Order sets were temporarily changed to unbundle the CT, CTA, and perfusion study order set.

Data collection and processing

The data was collected and managed using REDCap, a secure, web-based application that supports data capture for research studies (https://www.project-redcap.org/). No sample size was calculated as the entire eligible population during the designated study periods was included. Clinical characteristics, demographic characteristics, and imaging studies were summarized with frequency counts and percentages. Comparisons of pre- and post-implementation values were evaluated using X2 tests for binary variables. All statistical tests are two-sided, and a p-value of <0.05 indicates statistical significance. Data analyses were conducted using Analyse-it Version 4.95.4 (Analyse-it Software, Leeds, UK). 

## Results

During the pre- and post-implementation study periods, 4,574 and 3,973 CT studies were performed on 2,777 and 2,600 patient visits. All CTs were included in the final analysis. Pre-implementation, the median age was 58 years [IQR 41-73], and 1,259 (45%) subjects were males. During the post-implementation, the median age was 60 years [IQR 42-74], and 1,153 (44%) subjects were males. The median length of stay (p=0.013) and time to CT (p<0.001) both decreased during the post-implementation period. Complete demographic and clinical data for these subjects are shown in Table 1.

**Table 1:** Demographics Characteristics of Subjects IQR: interquartile range

	Pre-Implementation		Post-Implementation		
	(n=2,777)		(n=2,647)		P Value
Age (median (IQR))	58	(41-73)	60	(42-74)	0.025
Gender (n,%)					0.556
Male	1259	45%	1259	48%	
Female	1518	55%	1468	55%	
Insurance Status (n,%)					0.133
Commercial	997	36%	962	36%	
Governmental	1729	62%	1616	61%	
Uninsured/Unknown	51	2%	69	3%	
Race (n,%)					0.807
White	1865	67%	1775	67%	
Black	257	9%	268	10%	
Asian	98	4%	90	3%	
Other/Multiracial	557	20%	514	19%	
Length of Stay in Minutes (median (IQR))	409	(287-678)	389	(262-688)	0.013
Arrival to First CT Scan in Minutes (median (IQR))	143	(94-216)	127	(82-194)	<0.001
Visit Type					0.012
Treat and Release	1658	60%	1668	63%	
Admitted	1119	40%	979	37%	
Total ED Volume	9495		10394		

As seen in Table 2, During the pre-implementation period, 56 (1%) non-contrast studies were followed by a repeat study of the same body part with contrast within 1-24 hours versus the post-implementation period, whereas 55 (15%) non-contrast studies were followed by a contrast study. Among repeat studies, there was an increased incidence of abdomen and pelvis imaging and chest imaging during the post-implementation period (p=0.003). In the pre-implementation period of the abdomen and pelvis studies, all five repeat studies were with i.v. contrast only. During the post-implementation period, 11 studies (61%) were repeated with i.v. contrast only, seven (39%) were repeated with i.v. and oral contrast.

**Table 2:** Incidence of Repeat Computed Tomography Scans With Contrast

	Pre-Implementation		Post-Implementation		
	(n=56)		(n=55)		P Value
Body Part (n,%)					0.003
Head	50	89%	34	62%	
Neck	0	0%	0	0%	
Spine	0	0%	0	0%	
Chest	1	2%	3	5%	
Abdomen and Pelvis	5	9%	18	33%	
Extremity	0	0%	0	0%	

As shown in Table 3, the overall proportion of non-contrast imaging was higher post-implementation. Imaging of the abdomen and pelvis was also higher post-implementation (p<0.001). Additionally, more visits in the post-implementation period were discharged from the ED (p=0.012).

**Table 3:** Distribution of Computed Tomography Scans Ordered

	Pre-Implementation		Post-Implementation		
	(n=4,574)		(n=3,973)		P Value
Body Part (n,%)					<0.001
Head	1638	36%	1402	35%	
Neck	267	6%	90	2%	
Spine	397	9%	414	10%	
Chest	679	15%	532	13%	
Abdomen and Pelvis	1551	34%	1488	37%	
Extremity	42	1%	47	1%	
Contrast Type (n,%)					<0.001
No Contrast	1874	41%	2834	71%	
Oral Contrast	12	0.3%	37	1%	
IV Contrast	2476	54%	983	25%	
Oral and Intravenous Contrast	212	5%	119	3%	

## Discussion

This study aimed to investigate the effects of a global ICM shortage on the ordering patterns of emergency medicine (EM) physicians. The results of this study demonstrated that the actions taken to mitigate the impact of a worldwide ICM shortage resulted in an increased percentage of non-contrast imaging ordered. The incidence of repeat studies with ICM was highest for head, and abdomen and pelvis CTs. Lastly, there was a decrease in ED LOS and arrival to the first CT scan.

This study is unique in that there are no reported studies on the effects of a regional or global ICM shortage on the ordering patterns of EM clinicians. A related study by Allen et al. during routine availability of ICM described successful mitigation tactics to reduce i.v. contrast use by 50% for interventional radiology, interventional neurology, interventional cardiology, and electrophysiology [5]. Numerous statements and strategies have recently been published on addressing the ICM shortage [1, 3, 4, 7, 8]. The American College of Radiology (ACR) Committee on Drugs and Contrast Media released strategies to reduce the use of i.v. contrast. These strategies include the use of alternative studies such as non-contrast CT, MRI with or without gadolinium-based contrast media, ultrasound with or without ultrasound contrast agents, nuclear medicine, or positron emission tomography (PET) - CT scan [4]. Additional institution-wide guidance from the ACR includes strategies such as repackaging single-use vials into smaller aliquots to reduce waste, and working with other departments to prioritize limited supply usage. Many of the recommendations instituted by Northwell Health’s emergency medicine service line and the system’s Chief Medical Office incorporated suggestions and strategies discussed in the literature. While an abundance of strategizes and information was made available, we also urged our providers to rely on clinical decision-making tools.

In the current study, we identified a change in the overall ordering pattern of CT imaging. During the post-implementation period, a higher percentage of non-contrast CT scans were ordered (71% vs. 41%). There was also an overall decrease in the percentage of CT scans with i.v. contrast only (25% vs. 54%) and CT scans with oral with i.v. contrast ordered (3% vs. 5%). The decrease in CT scans using i.v. contrast and oral with i.v. contrast signifies that the mitigation strategizes worked.

**Figure 1:**
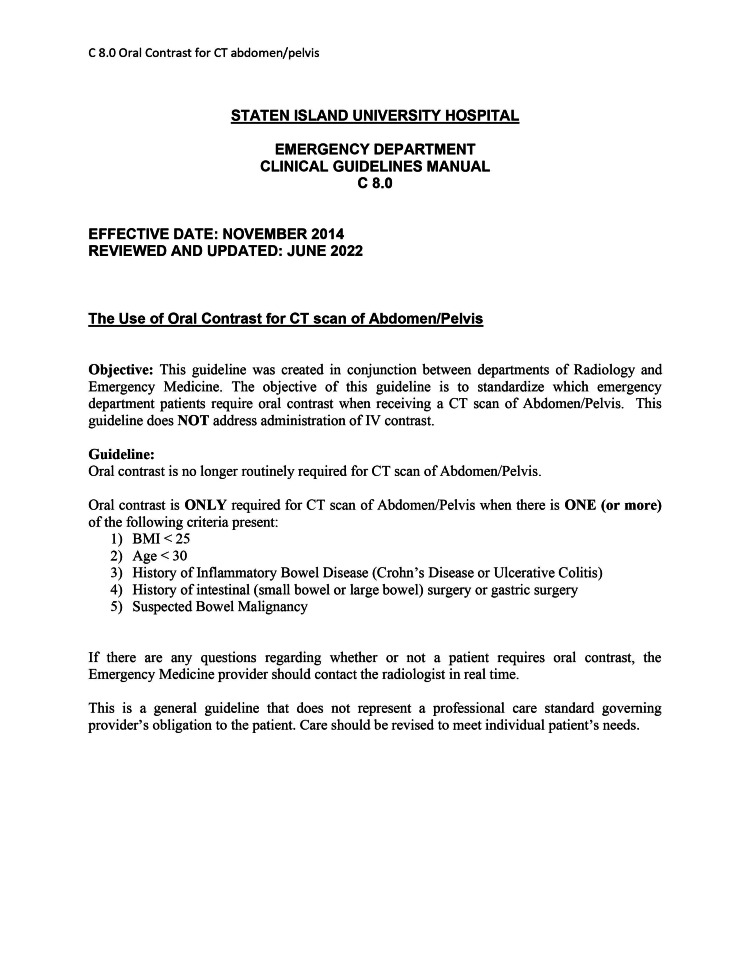
Staten Island University Hospital Clinical Guideline for the Use of Oral Contrast for Computed Tomography scan of Abdomen and Pelvis

Furthermore, our department has preexisting clinical guidelines for standardizing oral contrast in CT imaging of the abdomen and pelvis (Figure 1, Appendices). This guideline was created in conjunction with the institution’s radiology department to reduce unnecessary use of contrast and throughput time in the ED. Our physicians continued utilizing these oral-contrast guidelines during the study period. During the post-implementation period, there was an overall decrease in oral contrast used. While there was an increase in CT scans with oral contrast only during the post-implementation phase (1% vs. 0.3%), combining the total number of CTs with both oral and i.v. contrast, as well as oral-only CTs of the abdomen and pelvis, there was a decrease during the post-implementation phase (224 vs. 156 studies). The slight increase in CT scans with oral contrast only may have been due to the anecdotal perception that the ICM shortage was mainly of i.v. contrast, but given the overall numerical decrease, our interventions were largely successful.

There was a statistically significant difference between groups regarding the incidence of repeat CT scans with i.v. contrast. The increase in repeat abdomen and pelvis imaging (33% vs. 9%) during the post-implementation phase is likely due to a limited or non-diagnostic CT without contrast.

Only three (5%) repeat chest CT examinations were done with i.v. contrast in the post-implementation period. Although we did not review the indications for the repeat CT with contrast, this implies that the change in our trauma protocol to perform non-contrast CT was largely successful. Furthermore, there was a decrease in the number and percentage of chest CTs ordered during the post-implementation (13% vs. 15%). This decrease may be attributed to a reliance on a clinical decision and alternative imaging modalities to evaluate for pulmonary embolism. Fewer repeat CTs of the head with i.v. contrast scans were ordered (62% vs. 89%) during the post-implementation period. This reduction may be due to the intervention by our administration regarding the initial workflow for stroke patients. Unbundling the CT, CTA, and perfusion studies from the initial stroke order set and discussing with the on-call neurologist the need for CTA or computed tomography perfusion (CTP) before ordering all likely impacted this variable.

Most institutions have a policy of waiting for laboratory results, specifically for renal function, before administering i.v. contrast. Our study found that the median ED LOS and arrival time to initial CT significantly decreased during the contrast shortage period. This decrease may result from not waiting for laboratory results since fewer studies with i.v. contrast were ordered. Although not directly measured during this study, there are other potential benefits to ICM mitigation. These include reduced risk of allergic and systemic contrast-mediated reactions reported in the literature [9].

The limitations of our study include that it was conducted in a single institution with a sample size of 53,774 patient encounters. Although we took steps to ensure decreased use of ICM during this time, our institution maintained an adequate supply for all patient care required. Smaller or community institutions might require stricter measures to reduce their ICM utilization. Our study did not consider patients who may not have received a CT scan due to the contrast shortage. Most likely, these patients received a non-contrast CT scan or an alternative imaging examination.

Additionally, we did not conduct patient follow-up to identify if there was clinically significant missed pathology on the non-contrast-only CT scans. Anecdotally, we are unaware of any adverse outcomes through our performance improvement process, and 72-hour ED return review policy. Further research should evaluate the impact of ICM shortages on patient outcomes, compare the success of contrast mitigation tactics, assess the financial effect, and determine the impact of ICM shortages on other radiology imaging modalities.

## Conclusions

The primary goal of this study was to describe the effects of an ICM shortage on the ordering patterns of EM physicians. The results of this study demonstrated that the actions taken to mitigate the impact of a worldwide ICM shortage resulted in an increased percentage of non-contrast imaging ordered. More non-contrast CT scans were ordered. However, there was also an increase in repeat imaging with ICM material. The median ED LOS and arrival time to initial CT significantly decreased during the contrast shortage period. This decrease may result from not waiting for laboratory results since fewer studies with i.v. contrast were ordered. Further research should evaluate the impact of ICM shortages on patient outcomes, compare the success of contrast mitigation tactics, assess the financial effect, and determine the impact of ICM shortages on other radiology imaging modalities.

## Appendices

**Table 4:** Non-Contrast Computed Tomography Protocols for Body, Chest, Neurology and Pediatrics

Area of Body	Perform Non-Contrast Computed Tomography Study (Historic non-intravenous contrast CT exams)	Perform Non-Contrast Computed Tomography Study (Studies converted to non-intravenous contrast exams due to the iodinated contrast shortage)
Body	Flank pain/stone hunt Retroperitoneal bleed Low risk trauma (e.g., fall on anticoagulation therapy) Adrenal lesion	Diverticulitis > 200 lbs. Appendicitis > 200 lbs. Suspected hernia (palpable) Non-specific abdominal pain > 200 lbs. Oncology- testicular, lymphoma, myeloma Prostate cancer work-up: non-contrast CT or pelvic MR Renal lesion if suspect AML Note: MRI for liver lesion, enterography and renal lesion (if do not suspect AML) PET/CT can also be used for lymphoma
Chest/Cardiac	Acute parenchymal disease (e.g., pneumonia) HRCT, ILD, fibrosis Pulmonary nodules Metastatic disease Lung cancer screening Asbestos Virtual Bronchoscopy Calcium score	
Neuro	Trauma without suspected vascular injury Change in mental status Congenital anomaly Most screening head CTs Multiple indications that do include stroke, vascular tumor or abscess Neck CT for sialoliths or foreign body Spine degenerative disease	Screening exams that have low suspicion for cancer or infection Very low clinical likelihood of stroke, where CTA/CTP can be replaced by MRI/MRA If patient is outside treatment window or not a candidate for intervention, do MRI/MRA instead of CTA/CTP after the non-contrast head CT
Pediatric	Follow Body, Chest and Neuro protocols above except for low-risk body trauma which will get IV contrast Note: If appendicitis, start with ultrasound. Do IV CT if ultrasound is inconclusive.	
